# Patient and provider perspectives on using goal attainment scaling in care planning for older adults with complex needs

**DOI:** 10.1186/s41687-022-00445-y

**Published:** 2022-04-13

**Authors:** Catherine A. Clair, Shana F. Sandberg, Sarah H. Scholle, Jacqueline Willits, Lee A. Jennings, Erin R. Giovannetti

**Affiliations:** 1 Johns Hopkins Bloomberg School of Public Health, 615 N Wolfe St, Baltimore, MD 21205 USA; 2 NORC at the University of Chicago, 4350 East-West Highway, Bethesda, MD 20814 USA; 3 National Committee for Quality Assurance, 1100 13th St, NW, Washington, DC 20005 USA; 4 Reynolds Section of Geriatric Medicine, University of Oklahoma Health Sciences Center, 1122 N.E. 13th Street, ORB 1200, Oklahoma City, OK 73117 USA; 5 MedStar Health Economics and Aging Research Institute, MedStar Health Research Institute, 5601 Loch Raven Boulevard, Baltimore, MD 21239 USA

**Keywords:** Qualitative, Goal-based care, Person-centered care, Goal attainment scaling

## Abstract

**Background:**

Assess the feasibility of using goal attainment scaling (GAS) in care planning for older adults with complex needs. GAS is an individualized approach to goal setting and follow up using a quantified scale. To date, little is known about the feasibility of GAS among this population.

**Methods:**

We conducted a qualitative study with a sample of 28 older adults and 23 providers from diverse settings to evaluate the value and challenges of this approach. We conducted semi-structured interviews and iteratively coded and analyzed interview transcripts for themes related to value, challenges, and implementation.

**Results:**

Most older adults and providers reported that the GAS approach added value to the care encounter. GAS supported collaboration and patient accountability for their goals, though it could be demotivating to some patients. Some older adults and providers noted that GAS could be confusing and that it was uncomfortable to talk about negative outcomes (i.e., the − 2 and − 1 boxes of the scale). Factors that facilitated implementation included using visual copies of the GAS forms, having an established patient-provider relationship, practicing the approach, and having previous goal-related clinical training.

**Conclusions:**

GAS was feasible to implement across diverse settings, and, despite challenges, both older adults and providers reported that it added value to care planning encounters with the potential to improve delivery of person-centered care. Further efforts to demonstrate the applicability and benefit of this method for older adults are warranted, particularly to address implementation of the approach.

## Background

There is broad agreement that patient goals and priorities should guide care and quality measures used to evaluate care [1–3]. For older adults with multiple chronic conditions and functional limitations, clinical guidelines have pointed to the importance of providing goal-based care [4, 5]. For this complex population, goal-setting has shown to reduce patient-reported treatment burden and receipt of unwanted care and correlates with greater physical and social wellbeing and care satisfaction [6, 7]. Additionally, the Centers for Medicare and Medicaid Services (CMS) supports aligning care with patients’ goals as demonstrated by the “Meaningful Measures” initiative, which calls for quality measures where “care is personalized and aligned with patient’s goals” [8].

Despite agreement on its importance, there is wide variation in approaches to eliciting and documenting goals [9, 10]. Without a consistent method for documenting goals and tracking progress for complex patients, professional health and social care providers^1^ may have no way to evaluate whether care is helping patients achieve the outcomes important to them. A standardized approach to patient-centered goal elicitation, documentation, and progress tracking could help both providers and health systems ensure patients with complex conditions achieve what matters most to them.

**Fig. 1:**
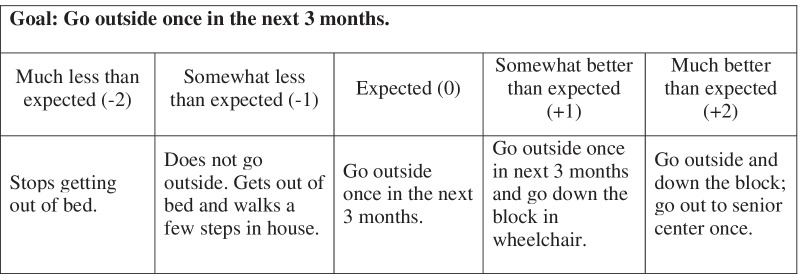
Example of scaled goal using goal attainment scaling

Goal attainment scaling (GAS) offers a structured approach for goal setting and measuring goal progress that could support measurement and monitoring. In addition to facilitating patient-centered goals, a key feature of GAS is that goals can be customized to the individual, but their progress can be tracked in a standardized way resulting in a quantitative score comparable across individuals [11]. The GAS process involves patients and providers jointly setting a goal and defining a set of possible outcomes along a 5-point scale ranging from “worse than expected” to “better than expected”. A numerical weight from − 2 to + 2 is assigned to each possible outcome (Fig. 1). GAS allows patients to articulate a goal that is important to their self-defined well-being and work collaboratively with their provider to develop a care plan that allows scaled achievement of that goal.

At follow-up, the patient and their provider discuss the individual’s progress and determine which outcome most closely matches what the individual achieved in the time since their previous visit.

GAS has been used in various settings, including hospital [12–14], primary care [15], and physical rehabilitation [16], and with diverse populations of older adults with complex needs, including older adults with multiple chronic conditions [15], individuals with cognitive disorders [17] and dementia [18], and older adults with functional limitations [19]. Toto et al. [15] showed that the GAS approach was feasible in geriatric primary care and aided in setting meaningful goals. In a study in dementia care management with 101 patients, Jennings et al. [18] likewise found that the approach was feasible, and 74 percent of participants achieved their goals. Similar results for goal achievement were seen in Waldersen et al. [19], with 74 percent of 226 older adults with functional limitations attaining their goals.

Despite its promise, studies using GAS with older adults have often been limited in sample size or to single site evaluation [15, 18, 20–22],many have solely focused on populations facing a single acute diagnosis [18, 23–25]. Several studies have highlighted the additional training, trust-building, and time needed to administer GAS [22, 24], suggesting that the integration of GAS into routine care may prove challenging. To our knowledge, studies have yet to assess the benefits and challenges of integrating GAS into primary care or complex care management, nor have they conducted qualitative analysis to understand the experience of both providers and older adult patients using GAS in such clinical settings.

To address this gap, we conducted a pilot study to understand the feasibility of implementing GAS in routine clinical care across seven clinical sites in the United States [26]. In this pilot, GAS was implemented with 184 older adult patients with multiple chronic conditions and functional disability (10–59 patients per site) and 33 providers. Results from the study found that most of the individuals using GAS met their goal (74%). Providers rated the GAS approach as useful for providing patient care, but the study did not address the older adults’ and providers’ experience with the process. To fill this gap in the literature, we report on the experiences of older adults and providers and summarize the themes that emerged from in-depth, qualitative interviews that covered both perceived value and challenges using GAS in care planning.

## Methods

### Study design, setting and participants

Seven sites participated in a learning collaborative designed to improve the quality of care for older adults with complex conditions by incorporating GAS into routine care (Table 1). Three of the organizations were private health insurers, two were integrated health care systems, and two were geriatric home-based primary care practices; they varied in geographic region, organization structure, specific population targeted, and number and type of providers who participated (see Table 1) [26].

**Table 1:** Participating sites

Location	Type of organization	Settings of care	Patient population served	Integration of care with primary care	Clinicians interviewed	Patients interviewed
Ohio	Case management for Medicare-Medicaid Managed Care Plan^1^	Telephone Home	Low-income older adults with long-term care needs	Case managers work with networked providers but are not integrated into primary care practice	4	1
Wisconsin	Case management program in Medicare-Medicaid Dual Eligible Special Needs Plan^2^	Home	Low-income older adults with long-term care needs	Case management team works with networked providers but are not integrated into primary care practice	2	7
Michigan	Transitional-care case management program in Medicare Advantage Plan^3^	Skilled Nursing Facility (SNF) Telephone	Older adults with complex medical conditions	RN case managers work on site at SNF to facilitate transition to community	5	7
California	Accountable care organization (ACO)^4^ case management program	Clinic Telephone	Older adults with complex medical conditions	Case managers work with primary care practice and ACO-affiliated specialists to coordinate care	4	5
Oregon	Medical home case-management program in integrated provider-health plan network^5^	Clinic Home Telephone	Home-bound older adults	Case management team is part of medical home and integrated into primary care practice	4	2
California	Geriatric home-based primary care	Home	Home-bound older adults	Physician and nurse practitioner provide primary care in the home	1	2
Texas/Michigan	Geriatric home-based primary care	Home	Home-bound older adults	Physician and registered nurse provide primary care in the home. Home care and hospice provider offices are co-located	3	4

^1^Private health insurer funded by the U.S. federal government and enrolls both Medicare and Medicaid beneficiaries. Medicaid payments are realized through contracted arrangements between state agencies and managed care organizations that accept a set per member per month payment for services

^2^Private health insurer funded by the U.S. federal government that enrolls individuals entitled to both Medicare and medical assistance from a state plan under Medicaid

^3^Medicare-approved private health insurance company that provides bundled services (Parts A, B, and D) of Medicare

^4^Group of doctors, hospitals, and other health care providers that provides coordinated care to Medicare patients. Accountable care organizations tie provider reimbursements to quality metrics and reductions in the cost of care

^5^A private (non-government) health system with integration of the delivery of health care, including integration of the electronic health record. Serves patients with private and public insurance

The learning collaborative occurred from July 2016 through July 2017. Providers at each site participated in an in-person training (1.5 days) on GAS and implemented GAS in their practice as a quality improvement activity. The learning collaborative included knowledge sharing across the participating organizations in monthly hour-long telephone meetings and an in-person conference at the end of collaborative.

Between March and July 2017, we conducted qualitative interviews with patients and providers to understand their perspectives on setting goals, measuring progress, and achievement of outcomes using the GAS approach. The Advarra Institutional Review Board (Columbia, Maryland) approved this study.

For the qualitative interviews with patients, we recruited 28 individuals from 184 older adults who participated in the larger study [26] based on their willingness to meet with researchers and share experiences [27]. Our sampling approach was a multi-step opt-out process for the older adults. Providers at the participating organizations approached older adults to gauge their interest in participating in an interview. If the older adult expressed interest, the researchers contacted the older adult to confirm their interest and schedule the interview during a 3–4 day window when the study team was on-site. We used two sample, two-tailed t-tests and chi-square tests to compare the characteristics of interviewed patients to patients who did not participate in interviews.

We conducted qualitative interviews with 23 providers. At three of the sites, we interviewed all providers participating in the pilot and at the remaining 4 sites a smaller subset of providers based on their willingness to be interviewed and availability during the site visit.

### Data collection and analyses

Separate semi-structured interview guides were developed for older adults and providers. The guides included questions related to the process of using GAS and the overall experience with the approach.

Prior to the interview, all participants (older adults and providers) were provided with an information sheet describing the study and the interview. At the beginning of the interview, the researchers reviewed the information sheet and obtained informed consent prior to conducting the interview. All participants provided verbal consent to participate in our study.

#### Interviews with older adults

At each site, two researchers trained in qualitative methods conducted interviews with older adults. Both researchers were present for each interview. Interviews lasted an average of 30 min and were conducted in the older adult’s usual care setting: in-clinic (n = 8) or at home, either in person (n = 11) or by telephone (n = 9). All older adults interviewed received a $50 gift card for their time and participation.

#### Interviews with providers

At each site, two researchers trained in qualitative methods conducted interviews with providers. Both researchers were present for each interview. Interviews lasted an average of 60 min, and generally took place in a clinic.

Interviews were digitally recorded and transcribed verbatim by a professional transcriptionist.

#### Analysis

Data analysis began in June and continued through September 2017. Although there was one-month overlap between data collection and analysis, our analysis did not inform the subsequent interviews (n = 3). We used an iterative approach in our analyses, allowing for emerging themes to shape subsequent analysis. To improve methodologic rigor and validity, the team conducted analysis utilizing three methodological principles: constant comparison (p. 210), negative cases (p. 89), and rival thinking (p. 211) [28].

The data analysis began with preliminary review of a sub-sample of interviews (N = 5) by the primary coder (CAC). An initial codebook was developed based on the interview guides and initial review of interviews. The coding team included two additional researchers. Inter-coder reliability for the coders was established with the same sub-sample of interviews. Inter-coder reliability was defined as applying the same code to a similar section or passage of the text.

The team independently coded the remaining transcripts using NVivo 11 Pro, a qualitative data management and organization software. The team met weekly to share observations, add new codes, and reach consensus on complex interview passages. The three methodological principles listed above informed these meetings: Team members were instructed and encouraged to compare that week’s coding to findings from previous weeks (e.g., constant comparison), to highlight instances in which data differed from the main themes (e.g., negative cases), and to acknowledge alternative interpretations (e.g., rival thinking).

The final codebook was developed after all transcripts were coded. Previously coded transcripts were reviewed with the final codebook to ensure that all codes were appropriately represented in the final analysis.

## Results

### Participant characteristics

On average, the older adult participants (N = 28) were 74 years of age (SD 8.81) with multiple chronic conditions (mean 3.71) and multiple activities of daily living (ADL) limitations (mean 2.79). The majority of older adults were female (67.9%), white (71.4%), and non-Hispanic (92.9%).

Older adults represented in our sample were similar to the population of the larger study in age, gender, race/ethnicity, chronic conditions, cognitive impairment and ADL limitations, with no statistically significant differences between the two groups on these characteristics (*p* > 0.05) (Table 2). Two interview participants were identified by their physician as having cognitive impairment but were still able to provide informed consent to interviews. Patients with cognitive impairment participated in the pilot with a friend or family caregiver who assisted in goal setting and monitoring goal achievement. However, interviews with caregivers of these patients were not included in this analysis.

**Table 2:** Comparison of patients interviewed to total sample

	Interview sample (N = 28)	Total sample (N = 184)
	Mean (SD)	Mean (SD)
Age^a^	74 (8.81)	74.41 (10.71)
Chronic conditions (out of 16)	3.71 (1.83)	3.78 (1.86)
ADL limitations (out of 6)	2.79 (1.77)	2.75 (2.09)

	N (%)	N (%)
Female	19 (68)	129 (70)
White	20 (71)	144 (78)
Hispanic	2 (7)	10 (5)
Cognitive impairment	2 (7)	36 (20)
Interview setting—Clinic	8 (29)	–
Interview setting—Home	11 (39)	–
Interview setting—Phone	9 (32)	–

^a^Ages above 89 recorded as 89

The 23 providers interviewed, comprised of registered nurses (10), nurse practitioners (4), social workers (6), physicians (2), and 1 patient navigator. Fourteen (60.9%) had more than 3 years of work experience at the participating sites. Of those with more than 3 years of experience, the average length of tenure was 5.4 years. Length of tenure was missing for 1 provider (social worker).

### Findings

Overall, most older adults and providers reported that using GAS added value to the care planning encounter, although some expressed challenges. Table 3 summarizes the key themes identified from interviews with additional quotes.

**Table 3:** Themes from interviews with older adults and clinicians using goal attainment scaling

	Quote(s)
Theme	
Goal attainment scaling allows for shared decision making with older adults and clinicians	*“When you’re so used to doing everything for yourself and you can’t do it anymore, so anyway, I think I was pretty low, thinking I don’t want to live like this. And she kind of brought out the idea of well, you’ve got so many skills, you know, why don’t you – and you talked about that – why don’t you do that? And so I did, with her encouragement”* – 82-year-old woman, Interview P25 *“I think that’s almost humble in, like, do you really – it was humbling to me always how little I know…And I’m always surprised, and that’s a good thing. I mean, it was humbling that no one had ever asked this person what she had wanted. And it was humbling to be with [name], like, well, I want to do a slideshow. I would never, you know, thought someone would have that capacity. That was, you know, it always makes me, like, as a clinician, it makes me stop and pause, and those were always good things, where I could just stop and pause.”* – Social worker, Interview C19
Goal attainment scaling supports communication and care planning	*“I think helpful because just the same thing, like having these conversations and…what comes out of the conversations is like ‘What do you need to make this happen, and how can we support you?’”* – Social worker, Interview C3
Goal attainment scaling adds accountability, which may motivate or demotivate patients	*“I told her, ‘I don’t want to set a timeline, because I don’t know if I can really reach that.’” You know, I don’t want to put a time limit on myself because, like I said, I – some days, I’m just really, really depressed, and I’ve been so sick…And lately I’ve been in a lot of pain, I feel, and re-injured my back. And, like, now I’m not focusing on nothing but trying to get this pain under control. So I don’t set a time limit on myself.”* – 67-year-old woman, Interview P1 “*When they see that there is so much potential for improvement without having to take these big leaps, I think that’s a motivating thing for them, too*.” – Registered nurse, Interview C12
Goal attainment scaling can be confusing	*“I would sit there and read it with them because some of them were kind of vague, you know…They were like – it was kind of like a tongue twister to them. They just didn’t understand, so I kind of like – that’s why I kind of read every one to them.”* – Physician, Interview C18
Goal attainment scaling includes scaling negative outcomes, which can be disconcerting for clinicians and patients	*“Well, we didn’t so much talk about what would happen to me if I didn’t reach my goals, but she has faith in me. She knows that I push myself and she knows I’ll reach them. She doesn’t doubt me.”* – 67-year-old woman, Interview P1 *“They’d be like, ‘Why are you asking me if it’s – like I’m going to meet this goal. Like, I’m going to do it. So why are you asking what would be a little bit worse? There’s no little bit worse. Like, this is – I’m going to do it,’ you know? And they were getting really frustrated by the scaling process.”* – Social worker, Interview C3
Facilitators and barriers	
A visual reminder	*“With my age to a lot of times I will forget…but with her coming in with the sheet, and this is what we said we’re going to do the last time I came in. I’m like, “OK.” Well, that makes me want to say, “Well, OK. Well, let me write this down here.” Or, “Let me stick it on my calendar*.*”*– 64-year-old man, Interview P15
Developing rapport	Interviewer: *Do you feel that she was comfortable talking with you about it?* 68-year-older woman: *Yes…Because she’s very easygoing, you know, to talk to and, you know…you can get along with her very well, you know, and she’ll communicate with you very easy.* (Interview P3)
Repeated use of the method	Interviewer: *Were you comfortable were you using the Goal Attainment Scale with patients?* Nurse practitioner: *Not comfortable at the beginning, but then the more you do it, very comfortable.* (Interview C17)
Previous training	*“It’s work that we were already doing. So it wasn’t, like, for some revolutionary, brand new topic or idea.”* – Registered nurse, Interview C5

#### Goal attainment scaling allows for shared decision making with older adults and providers.

Many older adults said they liked the collaborative nature of the process of working with their provider on setting a goal and using GAS to follow progress. For example, a 64-year-old woman described the goal-setting process as a process where “*we both had some input in it. That’s what was nice about it*” (Older Adult, Interview P16). Participants described goal setting in the care visit as a joint venture with their provider where both parties had a voice. A 77-year-old man said: “*I’ve compromised a little, she’s compromised a little*” (Older Adult, Interview P18).

Generally, providers viewed the process of goal setting and the GAS approach as supporting collaboration with their patients in the care visits. A social worker explained the positives of this collaborative effort, saying,

This is the [goal] that they are focused on because this is the one that they’ve had so much input on...this involves them having to have a discussion in what they want and how they hope to achieve it. (Provider, Interview C12)

Another provider said, "*It solidified the relationship. It solidified the transparency as far as what we were working towards, and what I heard*" (Provider, Interview C5).

#### Goal attainment scaling supports communication and care planning.

Older adults also explained how using the GAS approach differed from their usual process of working with their providers. One 75-year-old woman felt that she had *"more opportunities now to go after [her] goals*" when using the GAS approach (Older Adult, Interview P14). Participants also explained that the GAS approach was easier to understand as compared to the standard goal setting offered by the site. A 64-year-old man said,

Well, it’s a little easier for us. And well, you know, I don’t really -- I wasn’t really motivated, have the ambition, you know, to really say I know I should, but I didn’t. But with her and this program here, because she is like, “Well, OK, well...” And I do find out -- I have found out that in doing some of the things that we put down, there’s strength in me (Older Adult, Interview P15).

Several providers commented that the GAS was a useful method even when the older adult did not meet the goal they had set. In fact, some providers said that re-scaling the goal during follow-up encounters after challenges or setbacks was the most meaningful part of the GAS work. As a social worker explained, even when an older adult does not meet their goal,

It allows for a good conversation about…what the challenges were, and what kind of things can we help you to overcome those (Provider, Interview C3).

Several providers found that using GAS with their patients uncovered new goals and concerns. As one nurse described it:

So you go in and you’re like, ‘OK.’ In my head I think I kind of know what they’re going to pick. And they pick something completely different. That’s cool. I would have never pegged that (Provider, Interview C10).

A social worker commented that the GAS method “opened the door for discussion [of] things that they hadn’t even mentioned before” (Provider, Interview C14).

Providers also described the utility of the GAS method to inform patients' care plans. One nurse who visited older adults in a rehabilitation facility commented,

I was made more aware of the time spent at the bedside, how much you can gain from that conversation, how much information you can gain to make the right referrals and to set that patient up for even a greater success post-discharge (Provider, Interview C9).

#### Goal attainment scaling adds accountability, which may motivate or demotivate patients.

Many older adults described the process of goal setting as motivating. When asked why she wanted to continue with setting goals, a 68-year-old woman explained,

Because it kind of gives you incentive, you know, and a person being home all the time gets in a rut, you know, and do the same things every day, every day. And this kind of gives you a goal to do something (Older Adult, Interview P3).

A 58-year-old woman said,

Well, it’s [goal-setting] very important for me, because that’s -- that’s what makes me -- that’s what keeps me going (Older Adult, Interview P21).

Several older adults said they thought that other individuals could find the process to be motivating as well.

A 67-year-old woman described the GAS method this way:

It helps you focus because you’ve got the paper right there, and then you want to be able to write down that you walked and that you made some improvement, and it’s -- It reinforces -- It’s a positive reinforcement and I think that would help keep anybody just a -- you know, more motivated (Older Adult, Interview P6).

Despite their general positive feelings about the process, a few providers did express concern that patients might feel sad or guilty when they did not make expected progress. For example, when asked to compare the GAS method to usual practice, one social worker said,

I think maybe because this was like this process where it was documented, and it was this whole thing, it felt like she had somehow let us down by not achieving the goal (Provider, Interview C3).

Furthermore, some older adults said they felt bad when they did not meet the goal they had set for themselves. A 63-year-old woman commented that she did not like this method of working with her provider because,

I feel like I’m expected to do this, you know, after I say this is what I want to do…It makes me feel guilty when I can’t do what I said I wanted to do (Older Adult, Interview P17).

Another participant, a 67-year-old woman, worried prospectively about the potential outcomes of not reaching her goal and feeling negatively. She said, "*And then if my goal come, and I haven’t reached that goal, then I will feel bad"* (Older Adult, Interview P1).

#### Goal attainment scaling can be confusing.

Providers also worried about confusing patients with the process and the standardized scales involved with the GAS method. The 5-point GAS scale is a set of possible outcomes ranging from “worse than expected” to “better than expected”, with a numerical weight from − 2 to + 2 is assigned to each possible outcome. One nurse said,

I feel like the one thing that was a little bit confusing was the goal, and the starting at zero and the plus and the minus was slightly confusing to some people (Provider, Interview C13).

Some older adults also noted confusion with the GAS method for goal setting. A 63-year-old woman commented,

The whole thing is difficult...the questions, you know, making the goals and stuff. It’s always been hard for me to understand it since it came out, you know, and I really don’t like it (Older Adult, Interview P17).

Another example was mentioned by an 86-year-old man who described his confusion with understanding the direction he was supposed to be working towards with the scaling, which was resolved by a conversation with his provider.

86-year-old male: Sometimes I get confused on which way we’re going…but then when he tells me, I understand where we’re going. (Older Adult, Interview P20)

Interviewer: OK. So, it’s a little unclear -- so, it’s about how you’re scaling the goal to be better than expected but Dr. [Name] helps you kind of understand?

86-year-old male: Right. He explains it a little better and then I understand. (Older Adult, Interview P20)

#### Goal attainment scaling includes scaling negative outcomes, which can be disconcerting for providers and patients

Some providers commented on the issue of discussing negative outcomes (goal attainment that was less or much less than expected) with patients and worried about confusing or upsetting them. One nurse practitioner relayed an encounter to the interviewer:

If you got somebody that's really gung-ho... [and] you're bringing up, 'Well, what if you didn't do it?' [They] say, 'No, but I am going to...You're supposed to have confidence in me. You're supposed to think I can do this. Why are you asking me what it would look like if I don't do it?' So that was an interesting thing. That didn't happen all the time, but I think it happened a couple times (Provider, Interview C4).

#### Goal attainment scaling implementation facilitators and barriers

Across and within the seven sites, there were slight variations in how GAS was implemented. Providers noted several steps that made GAS easier to implement.

##### A visual reminder

At four of the seven sites, providers gave older adults a physical copy of their scaled goal. Several older adults at sites who provided a physical copy commented that they liked seeing their goal written out, and that the paper copy served as a physical reminder to focus them. As a 67-year-old woman said, *“it gave me something definite that I could see in black and white”* (Older Adult, Interview P6). In other interviews, older adults mentioned keeping the form in a visible place in their home—such as putting it on the refrigerator, in their calendar or appointment book, or by their bedside—in order to help them stay focused on meeting their goal.

A 63-year-older woman mentioned that had she been able to see the worksheet (which was present in the interview), it would have made more sense and she would have preferred to fill it out herself.

##### Developing rapport

Patients and providers said GAS was easier when they already had a relationship of rapport and trust.

Interviewer: What do you think makes it easy to talk to her?

82-year-old woman: Because I’ve developed trust, she’s developed trust. (Older Adult, Interview P25).

##### Repeated use of the method

Providers explained that the initial visit where the goal and the scaling was established was the longest and least comfortable as the GAS method was a new process to them. As providers continued to implement the GAS approach and became more comfortable, the time added to the length of the encounter decreased. A nurse described the experience, saying,

I became more comfortable with it. I mean, obviously, those first couple of times, I probably sounded like I was fumbling. But, towards the end...I was -- yeah, overall felt comfortable with it (Provider, Interview C9).

##### Previous training

Providers who had experience with goal setting or motivational interviewing expressed that GAS was more a continuation of their usual care.

Social worker: I mean this is where I kind of just tried to like weave it in because we normally have conversations with our patients about goals usually. So, you know, it would just kind of -- it didn’t feel that uncomfortable because I just sort of had the conversation that I would usually have around setting the goal (Provider, Interview C3).

## Discussion

This study suggests that that the use of GAS was feasible with older adults with multiple chronic conditions and functional limitations in various clinical settings, and older adults and providers found value in the process. Still, the GAS approach can be challenging, and participants identified key facilitators that may help to overcome these issues, such as using physical copies of the GAS form as a visual reminder, developing rapport in the patient-provider relationship, repeatedly using the method, and capitalizing on previous training like motivational interviewing. As with all qualitative research, the researchers bring their own experiences to data collection and data analysis. We acknowledge that the gender (female), race (White), ethnicity (non-Hispanic or Latino) and professional dynamics of our researchers may have influenced data collection. For example, our non-clinical backgrounds may have influenced how the providers tailored their responses (e.g., avoiding clinical jargon). In response, during data analysis, we attempted to maximize methodologic rigor in our research process [29].

Our qualitative findings expand upon and provide context for the findings of Giovannetti et al. [26]. Giovannetti et al. [26] found that, on a 10-point scale (1–10), providers rated GAS as high for usability on the three domains: Determining which services and supports to provide, helping patients achieve their goals, and helping patients track their progress [26]. While we might expect that a method for measuring goal progress and achievement would score well in the second and third domains, our qualitative interviews with providers may speak to the high scores for “determining which services and supports to provide”. Providers described GAS as adding value to the care encounter and supporting patient-provider communication and care planning. These perceived benefits may directly impact the provider’s decision-making process for determining the appropriate services and supports for their patient.

It is important to note, due to the study design and implementation of Giovannetti et al. [26], we cannot differentiate our findings specific to goal setting and findings specific to GAS. For many patients and providers, this was their first experience with both, challenging our ability to dissect these experiences. We recommend that future research efforts attempt to disentangle goal setting and GAS to improve our understanding of both separately as well as in combination.

The health care system often misses the opportunity to involve patients in their care, even less so to account for their diverse care preferences and goals [30]. The GAS approach in this study highlighted opportunities for older adults and providers to discuss what mattered most. Our findings of perceived improvements in shared decision-making, communication, and care plan development align with existing GAS literature [18, 22]. Javadi et al. [22] found that the use of GAS enabled providers to learn more about their clients’ priorities and health situation, which is supported by our findings from providers. For care managers of individuals with dementia and their caregivers, Jennings et al. [18] found that use of GAS improved provider understanding of what mattered to the patient and facilitated conversation regarding setbacks and limitations. Our study supports those findings from the provider perspective and supplements with the perspective of patients.

Our findings suggest that the GAS method may offer accountability, which was expressed by both older adults and providers. Accountability is a foundational aspect of patient activation and engagement, which is described as the patient’s “motivation, knowledge, skills, and confidence to make effective decisions to manage their health” [31]. Our study participants noted that the use of GAS increased their sense of accountability for their own goals. However, accountability was not universally deemed positive as some patients described it to be demotivating. The facilitators and barriers to implementation, such as established patient-provider rapport and previous clinical training to elicit goals, may offset these feelings of demotivation or fear of failure expressed by patients. To that end, our findings highlight that providers may benefit from additional training on GAS administration and motivational interviewing to better position them for successful goal-setting encounters with patients.

GAS is a tool that may offer value for both patient care and for evaluating quality of care. Using GAS, providers can have collaborative discussions with patients to identify the patient’s ideal outcome. By understanding and responding to the preferences and goals of the patient, providers may be better able to engage patients and caregivers, identify problems, and prescribe or recommend care more aligned with what matters most to them. With GAS, providers can measure these prioritized outcomes on a standardized scale and track progress over time. The standardized, yet personalized, methodology helps illustrate how well a provider or care team is helping individuals achieve the outcomes that matter most to them, a key pillar in The John A. Hartford Foundation and Institute for Healthcare Improvement Age-Friendly Health System initiative [32].

This study highlights areas of future research, particularly for integrating GAS into clinical care. Implementing GAS on a large scale [26] magnifies the challenges with integrating the approach. Considering barriers to implementation, providers in our study mentioned longer encounter times due to GAS, which improved with time but still increased the length of the care visit. Previous research has explored simplified approaches to GAS, including pre-worded goals and 3-point scaling [24, 33]. Time added to the encounter and provider buy-in for this approach need to be addressed moving forward, since longer encounter time often reduces provider revenue in the fee-for-service health care system. Further research into ways to improve the integration of goal-based care into existing financial reimbursement systems (i.e., replacing lower value care, developing an approach for billing this service as part of a value-based payment program or fee-for-service reimbursement system) can help reduce inefficiencies and address some of these concerns.

This study has a number of limitations. First, our findings were based on a small, convenience sample of older adults with multiple chronic conditions and functional limitations enrolled in care management programs. This sampling strategy was chosen to ensure availability and willingness to participate, as well as an adequate ability to communicate [34, 35]. Despite the limitations, this method resulted in a rich understanding of both the provider and patient experience [36, 37]. Second, our interview sample was predominantly female and white. Further research is needed to determine whether provider and patient perspectives would be similar when GAS is used with diverse patient groups.

## Conclusions

Overall, the GAS method is promising and requires training and preparation for all parties, including providers, patients, and family members. Given the potential benefit and the implementation learnings from this study, further efforts to demonstrate the applicability and benefit of the GAS approach for older adults are warranted. Such work should specifically address implementation issues on a larger scale.

## Data Availability

The data generated and analyzed during this study are not publicly available due to reasons of sensitivity. Data may be available upon reasonable request and with the permission of The National Committee for Quality Assurance.

## Appendix 1: Interview guide for providers

Process of using GAS approach

1. About how many older adults with functional limitations have you cared for in the past 12 months where a form like this was completed and reviewed during the visit?
2. Can you describe for me how you used the goal attainment scaling approach in the visit? Walk me through the process step by step.
3. Did using the goal attainment scaling approach add time to your average encounter with a patient?

Perceived value of GAS approach

4. When you began using this goal attainment scaling approach with patients, was it clear to you how this could help support patient achieve their goals and self-manage?
  - PROMPT: Did it work out the way you thought it would? Why or why not?
5. Can you tell me on a scale of 1-10 how helpful the goal attainment scaling forms have been for the following (10 being most helpful and 1 being least helpful)?
  - Helping patients track their progress
  - Determining which services and supports to provide to the patient
  - Helping the patient to achieve their goals
    - PROMPT to follow up on positive responses: Can you tell me why or how you think that works?

Discussing GAS with patients

6. How comfortable were you using the goal attainment scaling form with patients?
7. How comfortable were patients using the goal attainment scaling form?
8. Was there anything you did to make patients feel more comfortable using this form?
9. Would you want to continue to use the goal attainment scaling form with patients in the future? Why or why not?
10. Are there any changes you might recommend to the goal attainment scaling process to make it easier to use or more valuable?

Wrap up questions

11. Are there any ways that using goal attainment scaling process has surprised you? PROMPT: If yes, please explain
12. Are there any changes you might recommend to goal attainment scaling approach to make it easier to use or more valuable?

## Appendix 2: Interview guide for older adults

Process of using GAS

At your recent (or today’s) visit with [Provider], you talked about one or more goals using this worksheet. [*refer them to their last completed GAS worksheet*].

The goal you settled on was [insert goal from worksheet].

1. Is this important to you?
2. Is there another goal that would be more important to you?
3. Who decided what goal you would work on? You, [Provider], or someone else.
4. Was it difficult to decide which goal to work on?
5. What was it like using this worksheet to document the goal and outcomes?
6. What was it like tracking your progress towards achieving the goal using the worksheet?
7. Was the timeframe for attaining the goal too short, too long or just about right?

Experience of using goal attainment scaling in care provider conversations

8. How comfortable were you discussing the goal you documented with [Provider]?
  - Prompt: Was [care provider name] the right person to talk about this goal with? If not, who would you rather have discussed them with?
9. Did you feel that [Provider] was comfortable talking with you about it? If not, what made you think that?
10. Do you think [Provider] understands what is important to you?
11. Did the worksheet help [Provider] understand what is important to you? In what ways?
12. Did [Provider] make this goal part of your care plan? How?
13. Did you discuss how the goals would be achieved?
  - Prompt: How much help did the care provider give you in thinking about how to achieve your goals?
14. If you and [Provider] have talked about care plans in the past, how was it different using the worksheet? Can you give an example?

Wrap up questions

15. Do you want to use this worksheet again in the future with [Provider]? Why or why not?
16. Do you think that this worksheet could help other people to focus on care goals? What would you change the make it better?

## Footnotes

1 For the remainder of the manuscript, this term will be shortened to “providers”.

## References

[1] McGlynn EA, Schneider EC, Kerr EA (2014). Reimagining quality measurement. N Engl J Med.

[2] Reuben DB, Tinetti ME (2012). Goal-oriented patient care—an alternative health outcomes paradigm. N Engl J Med.

[3] Tinetti ME, Naik AD, Dodson JA (2016). Moving from disease-centered to patient goals-directed care for patients with multiple chronic conditions: patient value-based care. JAMA Cardiol.

[4] American Geriatrics Society Expert Panel on the Care of Older Adults with Multimorbidity (2012). Patient-centered care for older adults with multiple chronic conditions: a stepwise approach from the American Geriatrics Society: American Geriatrics Society Expert Panel on the Care of Older Adults with Multimorbidity. J Am Geriatr Soc.

[5] Care P-C (2016). A definition and essential elements. J Am Geriatr Soc.

[6] Kuipers SJ, Cramm JM, Nieboer AP (2019). The importance of patient-centered care and co-creation of care for satisfaction with care and physical and social well-being of patients with multi-morbidity in the primary care setting. BMC Health Serv Res.

[7] Tinetti ME, Naik AD, Dindo L, Costello DM, Esterson J, Geda M, Rosen J, Hernandez-Bigos K, Smith CD, Ouellet GM, Kang G, Lee Y, Blaum C (2019). Association of patient priorities-aligned decision-making with patient outcomes and ambulatory health care burden among older adults with multiple chronic conditions: a nonrandomized clinical trial. JAMA Intern Med.

[8] Meaningful Measures Hub | CMS. (2019, September 10). https://www.cms.gov/Medicare/Quality-Initiatives-Patient-Assessment-Instruments/QualityInitiativesGenInfo/MMF/General-info-Sub-Page

[9] Dunlay SM, Strand JJ (2016). How-to discuss goals of care with patients. Trends Cardiovasc Med.

[10] Hashim MJ (2017). Patient-centered communication: basic skills. Am Fam Physician.

[11] Kiresuk TJ, Sherman RE (1968). Goal attainment scaling: a general method for evaluating comprehensive community mental health programs. Community Ment Health J.

[12] Rockwood K, Stolee P, Fox RA (1993). Use of goal attainment scaling in measuring clinically important change in the frail elderly. J Clin Epidemiol.

[13] Stolee P, Awad M, Byrne K, Deforge R, Clements S, Glenny C, Day Hospital Goal Attainment Scaling Interest Group of the Regional Geriatric Programs of Ontario (2012). A multi-site study of the feasibility and clinical utility of goal attainment scaling in geriatric day hospitals. Disabil Rehabil.

[14] Stolee P, Rockwood K, Fox RA, Streiner DL (1992). The use of goal attainment scaling in a geriatric care setting. J Am Geriatr Soc.

[15] Toto PE, Skidmore ER, Terhorst L, Rosen J, Weiner DK (2015). Goal Attainment Scaling (GAS) in geriatric primary care: A feasibility study. Arch Gerontol Geriatr.

[16] Rushton PW, Miller WC (2002). Goal attainment scaling in the rehabilitation of patients with lower-extremity amputations: a pilot study. Arch Phys Med Rehabil.

[17] Bouwens SFM, van Heugten CM, Verhey FRJ (2008). Review of goal attainment scaling as a useful outcome measure in psychogeriatric patients with cognitive disorders. Dement Geriatr Cogn Disord.

[18] Jennings LA, Ramirez KD, Hays RD, Wenger NS, Reuben DB (2018). Personalized goal attainment in dementia care: measuring what persons with dementia and their caregivers want: personalized goal attainment in dementia care. J Am Geriatr Soc.

[19] Waldersen BW, Wolff JL, Roberts L, Bridges AE, Gitlin LN, Szanton SL (2017). Functional goals and predictors of their attainment in low-income community-dwelling older adults. Arch Phys Med Rehabil.

[20] Ashford S, Jackson D, Turner-Stokes L (2015). Goal setting, using goal attainment scaling, as a method to identify patient selected items for measuring arm function. Physiotherapy.

[21] Herdman KA, Vandermorris S, Davidson S, Au A, Troyer AK (2019). Comparable achievement of client-identified, self-rated goals in intervention and no-intervention groups: reevaluating the use of goal attainment scaling as an outcome measure. Neuropsychol Rehabil.

[22] Javadi D, Lamarche L, Avilla E, Siddiqui R, Gaber J, Bhamani M, Oliver D, Cleghorn L, Mangin D, Dolovich L (2018). Feasibility study of goal setting discussions between older adults and volunteers facilitated by an eHealth application: development of the Health TAPESTRY approach. Pilot Feasibility Stud.

[23] Grant M, Ponsford J (2014). Goal Attainment Scaling in brain injury rehabilitation: strengths, limitations and recommendations for future applications. Neuropsychol Rehabil.

[24] Krasny-Pacini A, Pauly F, Hiebel J, Godon S, Isner-Horobeti M-E, Chevignard M (2017). Feasibility of a shorter goal attainment scaling method for a pediatric spasticity clinic—the 3-milestones GAS. Ann Phys Rehabil Med.

[25] Tabak NT, Link PC, Holden J, Granholm E (2015). Goal attainment scaling: tracking goal achievement in consumers with serious mental illness. Am J Psychiatr Rehabil.

[26] Giovannetti ER, Clair CA, Jennings LA, Sandberg SF, Bowman A, Reuben DB, Scholle SH (2020). Standardised approach to measuring goal-based outcomes among older disabled adults: results from a multisite pilot. BMJ Qual Saf.

[27] Patton M (2020). Qualitative research & evaluation methods.

[28] Yin R (2015). Qualitative research from start to finish.

[29] Malterud K (2001). Qualitative research: standards, challenges, and guidelines. Lancet (London, England).

[30] Kogan AC, Wilber K, Mosqueda L (2016). Person-centered care for older adults with chronic conditions and functional impairment: a systematic literature review. J Am Geriatr Soc.

[31] Greene J, Hibbard JH (2012). Why does patient activation matter? An examination of the relationships between patient activation and health-related outcomes. J Gen Intern Med.

[32] Age-Friendly Health Systems (2020) Institute for Healthcare Improvement (IHI). http://www.ihi.org/Engage/Initiatives/Age-Friendly-Health-Systems/Pages/default.aspx

[33] Burnes D, Connolly M-T, Hamilton R, Lachs MS (2018). The feasibility of goal attainment scaling to measure case resolution in elder abuse and neglect adult protective services intervention. J Elder Abuse Negl.

[34] Palinkas LA, Horwitz SM, Green CA, Wisdom JP, Duan N, Hoagwood K (2015). Purposeful sampling for qualitative data collection and analysis in mixed method implementation research. Adm Policy Ment Health.

[35] Spradley JP (1979). The ethnographic interview.

[36] Miles MB, Huberman AM (1994). Qualitative data analysis: an expanded sourcebook.

[37] Polit DF, Beck CT (2010). Generalization in quantitative and qualitative research: myths and strategies. Int J Nurs Stud.

